# Knowledge of Thyroid Disease Manifestation and Risk Factors Among the General Population in the Tabuk Region of Saudi Arabia

**DOI:** 10.7759/cureus.56020

**Published:** 2024-03-12

**Authors:** Rawan S Alshahrani, Hyder Mirghani, Rahaf T Alharbi, Lama M Alenazi, Dalal L Albalawi, Ebtehal Mohammed D Alomrani, Layan Farhan N Alanazi, Abrar Ahmed A Aljayzani, Raghad D Alamri

**Affiliations:** 1 Internal Medicine, University of Tabuk, Tabuk, SAU; 2 Endocrinology, University of Tabuk, Tabuk, SAU

**Keywords:** saudi arabia, tabuk, risk factors, thyroid disease, knowledge

## Abstract

Background

Thyroid disorders are caused either by excessive or inadequate thyroid hormone production or by the enlargement of the thyroid gland. Various types of thyroid disorders exist, including primary (related directly to the gland itself), secondary (related to thyroid function), and other types. This study aimed to assess the knowledge of thyroid disease manifestation and its risk factors among individuals living in Tabuk, Saudi Arabia.

Methodology

An observational cross-sectional study was conducted among 400 adults living in Tabuk city of Saudi Arabia aged 18 years or above. Data was collected using an electronic questionnaire through a simple random sampling method. Data was then coded, entered, and analyzed using both descriptive and inferential statistical methods using IBM SPSS Statistics for Windows, Version 23.0 (Released 2015; IBM Corp., Armonk, New York, United States).

Results

A total of 403 participants were enrolled in this study. More than half (n=265, 65.8%) were females, and 188 (46.7%) were in the age group of 18-35 years. The educational level of the majority of them was a bachelor's degree (n=296, 73.4%). Hypothyroidism was the most common thyroid disorder among family members (n=51, 62.4%). Inadequate iodine intake was thought to be a risk factor for thyroid disorders, as mentioned by 276 (68.5%) participants. Exact 284 (70.5%) participants believed that females are more at risk of having thyroid disease. The mean total knowledge score was found to be 25.1±4.48 out of a total of 34. Exact 216 (53.6%) participants had good knowledge about thyroid disorders. The female gender had significantly higher levels of knowledge as compared to males (p-value=0.002) regarding the manifestation and risk factors of thyroid disorders. In terms of educational level, a bachelor's or above was found to be significantly associated with a higher level of knowledge (p-value=0.003).

Conclusion

A good level of knowledge and awareness about thyroid disorders was observed among more than half of the participants. Few knowledge gaps were identified regarding knowledge about symptoms of hypothyroidism and certain medications which might cause thyroid disorders.

## Introduction

Around 1.6 billion people are at risk of thyroid disorders, and its cases are reported in more than 110 countries. Thyroid disorders are one of the most neglected and underdiagnosed medical conditions. Further, there is a lack of knowledge regarding this significant problem among patients. In Saudi Arabia, only a limited number of studies have been conducted to assess the knowledge of thyroid disorders in the general population and its correlation with the diagnosis of the disease [1].

Thyroid disease is a prevalent disorder affecting millions of people worldwide. It refers to any dysfunction of the thyroid gland, including hyperthyroidism, hypothyroidism, and thyroid nodules. Thyroid disease can have significant health implications, including weight changes, fatigue, and mood disorders. Early detection and treatment are crucial in managing thyroid disease effectively [2].

Despite the high prevalence of thyroid disease, public awareness and knowledge of its manifestation and risk factors are limited, particularly in the Tabuk region of Saudi Arabia. This lack of knowledge can contribute to delays in diagnosis and treatment, leading to adverse health outcomes [2].

Studies conducted in different parts of the world have revealed poor public awareness and knowledge of thyroid disease. For instance, a study conducted in India found that only 13% of participants could identify the thyroid gland's location correctly. Similarly, a study conducted in the United States found that only 29% of participants were aware of the symptoms of hyperthyroidism [3].

In Saudi Arabia, few studies have been conducted to assess public awareness and knowledge of thyroid disease. A study conducted in Riyadh found that only 31.7% of participants had heard of thyroid disease. Another study conducted in Jeddah found that only 28.8% of participants had good knowledge of thyroid disease [4].

Therefore, there is a need for further research to assess the level of awareness and knowledge of thyroid disease manifestation and risk factors among the general population in the Tabuk region of Saudi Arabia. The proposed research project aimed to investigate the level of public awareness and knowledge of thyroid disease, identify the factors that influence thyroid disease recognition and treatment, and develop effective strategies to improve public awareness of thyroid disease. The findings from this study can inform the development of effective educational programs to improve early detection and treatment of thyroid disease, ultimately improving health outcomes for the population in the Tabuk region [5].

Similarly, a study with 1000 participants reported varying levels of knowledge and perceptions of thyroid disease [6]. Hence, this study aimed to explore the level of knowledge of thyroid disease manifestation and its risk factors among the general population in the Tabuk region of Saudi Arabia.

## Materials and methods

Research design and setting

This was an observational cross-sectional study conducted among adults living in Tabuk city of Saudi Arabia aged 18 years or above.

Sampling techniques and sample size calculation

A simple random sampling method was employed. A sample size of 400 was estimated using the Qualtrics calculator (https://www.qualtrics.com/blog/calculating-sample-size/), with a confidence level of 95% [7].

Recruitment procedure

An online structured questionnaire was presented to the participants for data collection. Data collection was done from June 2022 to January 2023. An informed consent was obtained from the online questionnaire after explaining the study in a written paragraph.

Inclusion and exclusion criteria

Residents of Tabuk city, Saudi Arabia, aged 18 years and above and who agreed to participate took part in the electronic self-administered questionnaire. However, those who refused to participate in the electronic self-administered questionnaire and those who were living outside Tabuk were all excluded.

Data collection procedure

An electronic self-administered questionnaire was used. The questionnaire consists of five parts. The first part consisted of biographical data (nationality, residence, age, gender, marital status, employment, education level, and annual income). The second part had questions about knowledge regarding risk factors of thyroid disorder symptoms and risk factors of sepsis. The third part contained knowledge about the clinical picture of thyroid disease. The fourth part assessed the knowledge of prevention, and the last part asked about past medical history and family history. Each variable of the questionnaire was graded using a common grading scheme representing 2 points for the correct option, 1 point for the neutral option, and 0 points for the incorrect answer. After summing up the points, a score of 25 points or more out of 34 (75% correctly answered questions) was considered good knowledge about thyroid disorders among the participants.

Data analysis

Data was entered using the Microsoft Office Excel software Version 2016. The data was then transferred to IBM SPSS Statistics for Windows, Version 23.0 (Released 2015; IBM Corp., Armonk, New York, United States) for statistical analysis. Descriptive statistics were obtained to summarize data, synthesize, and report the variables. Numerical data was presented as mean±SD. For categorical variables, percentages and frequencies were used. Comparison between groups was made by the chi-squared test and Fisher's exact test. The p-value was considered statistically significant if it was less than 0.05.

Ethical considerations

Ethical approval was obtained from the Research Ethical Committee at the University of Tabuk (approval number: UT-309-148-2023), dated 14-09-2023.

## Results

A total of 403 participants were enrolled in this study. More than half (n=256, 65.8%) of the participants were females, and 138 (34.2%) were males. Exact 188 (46.7%) participants were in the age group of 18-35 years. The vast majority (n=386, 95.8%) were of Saudi Arabian nationality. Most of the participants (n=281, 69.7%) were from Tabuk in terms of residence. Exact 273 (67.7%) participants were married. The educational level of the majority of the participants (n=296, 73.4%) was a bachelor's degree. Exact 165 (40.9%) participants were employed. Annual income in Saudi riyal (SAR )for most of the participants (n=237, 58.8%) was less than 120,000 SAR (Table 1).

**Table 1:** Socio-demographic characteristics of the study participants (n=403) Data is presented as N and % SAR: Saudi riyal

Variable	Categories	Frequency	Percent
Gender	Male	138	34.2
	Female	265	65.8
Age (in years)	18-35	188	46.7
	36-53	180	44.7
	More than 53	35	8.7
Nationality	Saudi	386	95.8
	Non-Saudi	17	4.2
Residence	Outside Tabuk	122	30.3
	Inside Tabuk	281	69.7
Marital status	Single	102	25.3
	Married	273	67.7
	Divorced	16	4
	Widowed	12	3
Educational level	Illiterate	1	0.2
	Primary school	3	0.7
	Secondary school	16	4
	High school	87	21.6
	Bachelor or above	296	73.4
Employment status	Employed	165	40.9
	Unemployed	104	25.8
	Student	48	11.9
	Health practitioner	33	8.2
	Self-employed	24	6
	Retired	29	7.2
Annual income (SAR)	<120,000	237	58.8
	120,000-200,000	123	30.5
	>200,000	43	10.7

Exact 78 (19.4%) participants had a history of thyroid disease. Among those with thyroid disorders, 51 (65.4%) participants had hypothyroidism, 12 (15.4%) participants reported a history of hyperthyroidism, and eight (10.3%) participants mentioned a history of thyroid nodules. Moreover, 197 (48.9%) participants stated that they had at least one of their family members diagnosed with thyroid disorders. Hypothyroidism was the most common thyroid disorder among family members as mentioned by 123 (62.4%) participants. A total of 196 (48.6%) participants had previously done thyroid gland investigations. The reason for thyroid gland investigations was found to be routine checks as reported by 99 (50.5%) participants followed by a doctor's suggestion in 31 (15.8%) participants (Table 2).

**Table 2:** Past medical history and family history of thyroid disorders Data is presented as N and %

Question	Categories	N (%)
Have you ever had a thyroid disease?	Yes	78 (19.4)
	No	325 (80.6)
If yes, which type of thyroid disorder do you have?	Hypothyroidism	51 (65.4)
	Hyperthyroidism	12 (15.4)
	Thyroid nodules	8 (10.3)
	Thyroid cancer	0 (0)
	I don't know	7 (9)
Has anyone in your family been diagnosed with a thyroid disorder?	Yes	197 (48.9)
	No	206 (51.1)
If yes, which type of thyroid disorder?	Hypothyroidism	123 (62.4)
	Hyperthyroidism	33 (16.8)
	Thyroid nodules	11 (5.6)
	Thyroid cancer	5 (2.5)
	I don't know	25 (12.7)
Have you ever done any thyroid gland investigations?	Yes	196 (48.6)
	No	207 (51.4)
If yes, what was the reason for the investigation of thyroid function?	Routine check	99 (50.5)
	Doctor's suggestions	39 (19.9)
	Symptoms of thyroid disease	31 (15.8)
	Neck swelling	14 (7.1)
	I don't know	13 (6.6)

Exact 233 (57.8%) participants thought that smoking is a risk factor for thyroid disease, while 272 (67.5%) believed that radiation exposure is a risk factor for thyroid disease. Inadequate iodine intake was thought to be a risk factor for thyroid disorders by 276 (68.5%) participants. Further, 284 (70.5%) participants believed that females are more at risk of having thyroid disease, while 301 (74.7%) participants don't know that certain medications such as amiodarone and Cordarone are considered to be a risk factor for thyroid disorders. Lithium intake was considered a risk factor for thyroid disorders by only 106 (26.3%) participants. Concerning knowledge about the clinical picture of thyroid disease, 302 (74.9%) participants knew that feeling cold and weight gain are common symptoms of hypothyroidism, and 277 (68.7%) participants also knew that feeling hot and weight loss are common symptoms of hyperthyroidism. Less than half (n=126, 31.3%) of the participants reported that diarrhea and constipation or stomachache can be symptoms of thyroid disease (Table 3).

**Table 3:** Knowledge regarding risk factors, clinical picture, and prevention of thyroid disorders Data is presented as N and %

Risk factors for thyroid disorders	Yes	No	Don't know/not sure
	N (%)	N (%)	N (%)
Do you think smoking is a risk factor for thyroid diseases?	233 (57.8)	50 (12.4)	120 (29.8)
Do you think radiation exposure is a risk factor for thyroid diseases?	272 (67.5)	38 (9.4)	93 (23.1)
Do you think insufficient or excess iodine intake is a risk factor for thyroid diseases?	276 (68.5)	27 (6.7)	100 (24.8)
Do you think pregnancy and postpartum period are risk factors for thyroid diseases?	154 (38.2)	100 (24.8)	149 (37)
Do you think females are more at risk of having thyroid diseases?	284 (70.5)	27 (6.7)	92 (22.8)
Do you think the medication amiodarone (known commercially as Pacerone, Cordarone, Advadarone, or Sedacoron) is a risk factor for thyroid diseases?	78 (19.4)	24 (6)	301 (74.7)
Do you think lithium intake is a risk factor for thyroid diseases?	106 (26.3)	24 (6)	273 (67.7)
Clinical picture of thyroid disorders			
Feeling cold and weight gain are common symptoms of having hypothyroidism	302 (74.9)	18 (4.5)	83 (20.6)
Feeling hot and weight loss are common symptoms of having hyperthyroidism	277 (68.7)	30 (7.4)	96 (23.8)
Do you think the neck lump can be a sign of thyroid diseases?	280 (69.5)	28 (6.9)	95 (23.6)
Do you think fatigue can be a symptom of thyroid diseases?	336 (83.4)	15 (3.7)	52 (12.9)
Do you think diarrhea, constipation, or stomachache can be symptoms of thyroid diseases?	126 (31.3)	83 (20.6)	194 (48.1)
Do you think bulging eyes can be a sign of thyroid diseases?	146 (36.2)	68 (16.9)	189 (46.9)
Do you think skin and nail changes or hair loss can be signs of thyroid diseases?	225 (55.8)	31 (7.7)	147 (36.5)
Prevention of thyroid disorders			
Do you think being away from soy food is one of the preventive ways for thyroid diseases in women?	116 (28.8)	57 (14.1)	230 (57.1)
Do you think early thyroid function tests can prevent the complications of thyroid diseases?	354 (87.8)	4 (1)	45 (11.2)
Do you think a well-balanced diet is essential to prevent thyroid diseases?	324 (80.4)	15 (3.7)	64 (15.9)

The mean total knowledge score was found to be 25.1±4.48 out of a total of 34. The differential knowledge score regarding the risk factors, clinical picture, and prevention was found to be 9.8±2.41, 10.5±2.53, and 4.8±1.03, respectively (Table 4).

**Table 4:** Knowledge scores regarding risk factors, clinical picture, and prevention of thyroid disorders

Score	Risk factors	Clinical picture	Prevention	Total knowledge
Mean±SD	9.8±2.41	10.5±2.53	4.8±1.03	25.1±4.48
Total possible score	14	14	6	34

**Figure 1:**
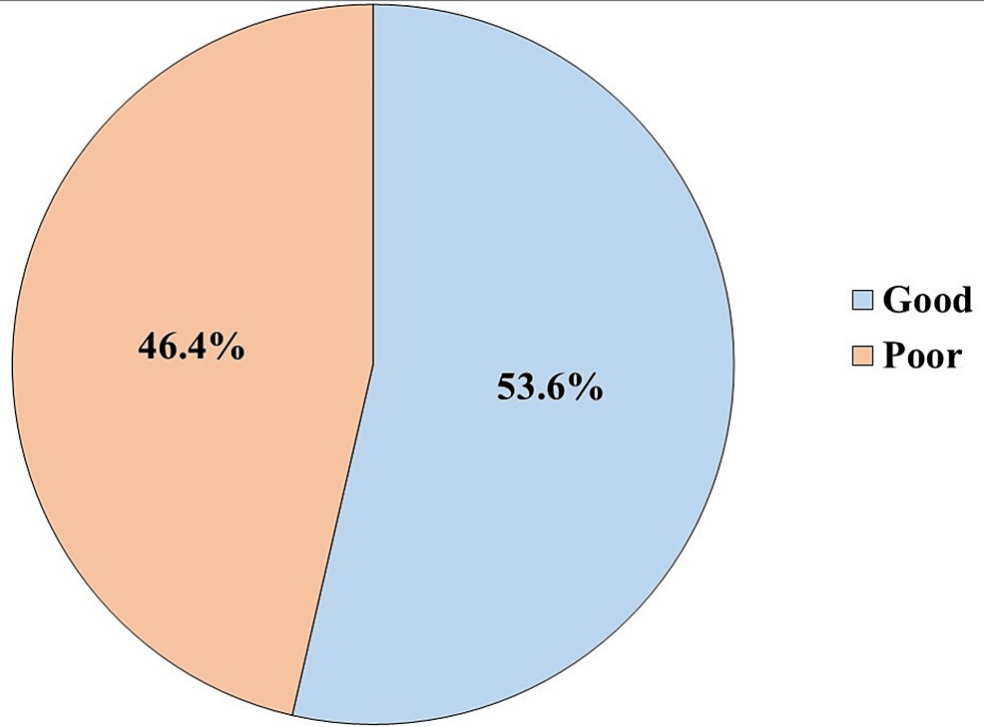
Level of knowledge regarding thyroid disorders

Exact 216 (53.6%) participants had good knowledge (Figure 1).

Regarding the association between certain variables and level of knowledge about thyroid disorders, gender was found to be significantly associated with the level of knowledge about thyroid disorders (p-value=0.002) with the female gender tending to have higher levels of knowledge compared to males (59.2% vs. 42.8%). A significant association was found between nationality and level of knowledge about thyroid disorders (p-value=0.011) as Saudi Arabians had a relatively higher level of knowledge compared to non-Saudi Arabians. In terms of educational level, bachelor or above degree holders were found to be significantly associated with a higher level of knowledge compared to others (p-value=0.003). Being a healthcare practitioner, having a family history of thyroid disorders, and doing thyroid gland investigations were associated with a higher level of knowledge and awareness about thyroid disorders (p-value=0.019, 0.013, and 0.003, respectively) (Table 5).

**Table 5:** Factors associated with the level of knowledge regarding thyroid disorders F: p-value calculated using Fisher's exact test, other p-values calculated using the chi-squared test *: significant p-value <0.05 SAR: Saudi riyal

Variable	Categories	Level of knowledge		P-value
		Good	Poor	
Gender	Male	59 (42.8)	79 (57.2)	0.002*
	Female	157 (59.2)	108 (40.8)	
Age (in years)	18-35	196 (56.4)	82 (43.6)	0.203
	36-53	96 (53.3)	84 (46.7)	
	More than 53	14 (40)	21 (60)	
Nationality	Saudi	212 (54.9)	174 (45.1)	0.011*
	Non-Saudi	4 (23.5)	13 (76.5)	
Residence	Outside Tabuk	62 (50.8)	60 (49.2)	0.461
	Inside Tabuk	154 (54.8)	127 (45.2)	
Marital status	Single	57 (55.9)	45 (44.1)	0.315
	Married	147 (53.8)	126 (46.2)	
	Divorced	5 (31.3)	11 (68.8)	
	Widowed	7 (58.3)	5 (41.7)	
Educational level	Illiterate	0 (0)	1 (100)	0.003^F^*
	Primary school	0 (0)	3 (100)	
	Secondary school	8 (50)	8 (50)	
	High school	35 (40.2)	52 (59.8)	
	Bachelor or above	173 (58.4)	123 (41.6)	
Employment status	Employed	82 (49.7)	83 (50.3)	0.019*
	Unemployed	61 (58.7)	43 (41.3)	
	Student	29 (60.4)	19 (39.6)	
	Health practitioner	24 (72.7)	9 (27.3)	
	Self-employed	8 (33.3)	16 (66.7)	
	Retired	12 (41.4)	17 (58.6)	
Annual income (SAR)	<120,000	121 (51.1)	116 (48.9)	0.140
	120,000-200,000	66 (53.7)	57 (46.3)	
	>200,000	29 (67.4)	14 (32.6)	
Have you ever had a thyroid disease?	Yes	48 (61.5)	30 (38.5)	0.117
	No	168 (51.7)	157 (48.3)	
Has anyone in your family been diagnosed with a thyroid disorder?	Yes	118 (59.9)	79 (40.1)	0.013*
	No	98 (47.6)	108 (52.4)	
Have you ever done any thyroid gland investigations?	Yes	120 (61.2)	76 (38.8)	0.003*
	No	96 (46.4)	111 (53.6)	

## Discussion

Assessing the level of knowledge and awareness about thyroid disorders is important as higher levels of knowledge and awareness were associated with reduced prevalence and better management and disease prevention methods [1].

Nearly one-fifth (n=78, 19.4%) of the participants had a history of thyroid disease, and this percentage is considered to be significantly higher than that reported in the parallel study carried out in Jordan [8].

Hypothyroidism was found to be the most common thyroid disorder among family members as mentioned by more than half (n=123, 62.4%) of the participants, and this percentage was found to be lower than mentioned in the congruent study conducted by Kollerits et al., in which 93.1% individuals had hypothyroidism [9]. Also, an analogous finding was reported in a study [10].

More than half (n=233, 57.8%) of the participants thought that smoking is a risk factor for thyroid disease and this was found to be contradictory to that reported in the study conducted by Wiersinga [11]. Inadequate iodine intake was thought to be a risk factor for thyroid disorders by more than two-thirds (n=276, 68.5%) of the participants, and this is in contradiction to the study conducted by Kayes et al., in which the level of knowledge about the importance of iodine in the prevention of thyroid disorders was poor [12]. The previously mentioned results were consistent with the study [13].

The majority (n=284, 70.5%) of the participants believed that females are more at risk of having thyroid disease, and similar findings were reported in a parallel study [6]. Also, the majority (n=301, 74.7%) of the participants don't know that certain medications such as amiodarone and Cordarone are considered to be a risk factor for thyroid disorders as reported in the congruent studies [14,15].

Regarding knowledge about the clinical picture of thyroid disease, more than two-thirds (n=302, 74.9%) of the participants knew that feeling cold and weight gain are common symptoms of having hypothyroidism, and more than half (n=277, 68.7%) of the participants also knew that feeling hot and weight loss are common symptoms of having hyperthyroidism. Nearly one-third (n=126, 31.3%) of the participants reported that diarrhea and constipation or stomachache can be symptoms of thyroid disease. One-third (n=146, 36.2%) of the participants mentioned that they knew that bulging of the eye can be a sign of thyroid disease, and similar findings were found in parallel studies [16,17].

The mean total knowledge score was found to be 25.1±4.48 out of a total of 34. About half (n=216, 53.6%) of the participants had good knowledge about thyroid disorders. The percentage of participants with good knowledge was higher than the findings of the analogous studies in which lower levels of knowledge were reported [16,18].

Gender was found to be significantly associated with the level of knowledge about thyroid disorders with the female gender tending to have higher levels of knowledge compared to males, and this was found to be consistent with the findings in Alzahrani et al.'s study [5].

Further, a bachelor's or above educational level was found to be significantly associated with a higher level of knowledge compared to others. Being a healthcare practitioner, having a family history of thyroid disorders, and doing thyroid gland investigations were associated with a higher level of knowledge and awareness about thyroid disorders, and analogous findings were mentioned in studies [19,20].

Limitations

The main limitation of this study was that the data were collected via a self-reported survey through online networks which might be influenced by reporting bias. The cross-sectional nature of this study and the convenience sampling used cannot confirm the causal association between the compared variables. Selection bias was also a possibility.

## Conclusions

A good level of knowledge and awareness about thyroid disorders among more than half of the participants was observed. Few knowledge gaps were identified regarding symptoms of hypothyroidism and certain medications which might cause thyroid disorders. Gender, educational level, and family history of thyroid disorders were linked with increased levels of awareness and knowledge about thyroid disorders. Efforts should be directed towards raising the level of knowledge and awareness of the population about thyroid disorders. This could be attained through various mechanisms including community campaigns and social events. Media nowadays have a significant role in the distribution of information and knowledge, and this could be used as a powerful tool to raise knowledge levels through media programs.
